# Sociodemographic disparities in targeted therapy in ovarian cancer in a national sample

**DOI:** 10.3389/fonc.2023.1104630

**Published:** 2023-05-12

**Authors:** Saber A. Amin, Lindsay J. Collin, Juraj Kavecansky, Soko Setoguchi, Jaya M. Satagopan, Elisa V. Bandera

**Affiliations:** ^1^ Cancer Epidemiology and Health Outcomes, Rutgers Cancer Institute of New Jersey, New Brunswick, NJ, United States; ^2^ Department of Radiation Oncology, University of Nebraska Medical Center, Omaha, NE, United States; ^3^ Department of Population Health Sciences, Huntsman Cancer Institute, University of Utah, Salt Lake City, UT, United States; ^4^ Department of Hematology/Oncology, Kaiser Permanente Northern California, Walnut Creek, CA, United States; ^5^ Department of Medicine, Robert Wood Johnson Medical School, New Brunswick, NJ, United States; ^6^ Center for Pharmacoepidemiology and Treatment Science, Institute for Health, Aging and Health Policy Research, Rutgers University, New Brunswick, NJ, United States; ^7^ Department of Biostatistics and Epidemiology, Rutgers School of Public Health, Piscataway, NJ, United States

**Keywords:** epithelial ovarian cancer, targeted therapy, cancer health disparities, epidemiology - analytic (risk factors), cancer treatment

## Abstract

**Background:**

The treatment landscape for ovarian cancer has changed in recent years with the introduction of targeted therapies to treat patients with advanced disease. We investigated patient demographic and clinical factors associated with use of targeted therapies as a part of the first-line treatment for ovarian cancer.

**Methods:**

This study included patients diagnosed with stage I–IV ovarian cancer between 2012 and 2019 from the National Cancer Database. Information on demographic and clinical characteristics were collected and described using frequency and percent across receipt of targeted therapy. Logistic regression was used to compute the odds ratios (ORs) and 95% confidence intervals (CI) associating patient demographic and clinical factors with receipt of targeted therapy.

**Results:**

Among 99,286 ovarian cancer patients (mean age 62 years), 4.1% received targeted therapy. The rate of targeted therapy receipt across racial and ethnic groups over the study period was fairly similar; however, non-Hispanic Black women were less likely to receive targeted therapy than their non-Hispanic White counterparts (OR=0.87, 95% CI: 0.76–1.00). Patients who received neoadjuvant chemotherapy were more likely to receive targeted therapy than those who received adjuvant chemotherapy (OR=1.26; 95% CI: 1.15–1.38). Moreover, among patients who received targeted therapy, 28% received neoadjuvant targeted therapy, with non-Hispanic Black women being most likely to receive neoadjuvant targeted therapy (34%) compared with other racial and ethnic groups.

**Conclusions:**

We observed differences in receipt of targeted therapy by factors such as age at diagnosis, stage, and comorbidities present at diagnosis, as well as factors related to healthcare access—including neighborhood education level and health insurance status. Approximately 28% of patients received targeted therapy in the neoadjuvant setting, which could negatively impact treatment outcomes and survival due to the increased risk of complications associated with targeted therapies that may delay or prevent surgery. These results warrant further evaluation in a cohort of patients with more comprehensive treatment information.

## Background

Epithelial ovarian cancer is the most lethal gynecologic malignancy and the fifth leading cause of cancer-related mortality among women in the United States (U.S.) (1). The current standard-of-care for ovarian cancer includes optimal cytoreductive surgery in combination with platinum-based chemotherapy typically including a carboplatin/paclitaxel chemotherapy regimen (2). An estimated 70% of ovarian cancer patients will experience a cancer recurrence following first line treatment with surgery and chemotherapy, contributing to a dismal median progression-free survival of 12–18 months (3). With each subsequent relapse, many patients develop chemoresistance and stop responding to standard chemotherapy regimens (4). Due to the high risk of recurrence and low five-year survival, novel therapeutic options are desperately needed to improve ovarian cancer outcomes (5–7).

The emergence of targeted therapies (e.g., bevacizumab) in the past few decades has revolutionized the treatment landscape of various malignancies, including ovarian cancer (6, 8). In several clinical trials, bevacizumab—a monoclonal antibody targeting vascular endothelial growth factor combined with chemotherapy—was associated with improved progression-free survival and overall survival among ovarian cancer patients diagnosed with advanced stage disease (9–13). Bevacizumab was first introduced as a maintenance therapy to treat recurrent ovarian cancer; however, results from clinical trials led to changes to the clinical guidelines to offer bevacizumab as a primary ovarian cancer treatment in 2012, with its use increasing in clinical settings (10, 14, 15). The incorporation of bevacizumab into first line ovarian cancer treatment has been associated with a reduction of platinum-resistant recurrence (16). The use of bevacizumab is approved in combination with chemotherapy for patients with stage III or IV disease after initial surgical resection or in combination with chemotherapy for platinum-sensitive recurrent disease (17). The most recent approval was based on GOG-0218 (NCT00262847), a multicenter, randomized, double-blind, placebo-controlled, three-arm study evaluating the addition of bevacizumab to carboplatin and paclitaxel for patients with stage III or IV epithelial ovarian, fallopian tube, or primary peritoneal cancer following initial surgical resection, which showed improved survival for stage IV patients (17, 18). Bevacizumab is associated with an increase in risk of complications when used prior to surgery that can delay or prevent interval debulking and is cautioned for use prior to surgery by current National Comprehensive Cancer Network (NCCN) guidelines (2, 19).

Despite the changing treatment landscape of ovarian cancer, to our knowledge, there have been no studies to date that have investigated the associations of patient demographic and clinical characteristics of women diagnosed with ovarian cancer with receipt of targeted therapy in a population-based clinical setting. Borrowing trends from other cancer therapies, we expect that there may be differential use by common social, socioeconomic, and demographic characteristics that are proxies for barriers in access to quality cancer care in the U.S. Therefore, using population-based data from the National Cancer Database (NCDB), we explored the trends and patterns in the use of targeted therapy over time, as well as identified factors associated with the use of targeted therapy in ovarian cancer patients.

## Materials and methods

### Data source

The NCDB was used to identify ovarian cancer patients and extract information surrounding the demographic and clinical characteristics of interest. The NCDB is the largest hospital-based cancer registry in the U.S., which captures more than 70% of cancer cases diagnosed annually. It is a consortium of more than 1,500 Commission on Cancer-accredited programs in the U.S. and Puerto Rico. Current study was exempt from Institutional Review Board (IRB) review as the NCDB data is a de-identified data source.

### Study population

We identified women diagnosed with a first primary invasive stage I–IV ovarian cancer between 2012 and 2019, who were aged 18 years or older at the time of their diagnosis. We chose the year 2012 as the start of the study period because bevacizumab was first approved for use as a first-line therapy for ovarian cancer in 2012 and, prior to 2012, the use of targeted therapies in ovarian cancer was limited. The variable in NCDB that indicates receipt of targeted therapy does not include information on type of therapy (*e.g.*, bevacizumab, PARP inhibitors, PDL-1 inhibitors), so we did not restrict our cohort to those with specific indication for bevacizumab (*i.e.*, late stage diagnosis). We excluded patients with missing surgery status (n=1,541), missing chemotherapy (n=2,062), missing targeted therapy (n=316), patients who were missing information on race or ethnicity (n=2,498), and patients with a racial and ethnic group classification of ‘Other’ (n=2,241).

### Study outcome

The primary study goal was to identify factors associated with receipt of targeted therapy. Therefore, the study outcome was receipt of targeted therapy (yes, no).

### Patient demographic and clinical characteristics

The factors of interest included age at diagnosis (years), race and ethnicity (non-Hispanic Black [NHB], non-Hispanic White [NHW], Asian, Hispanic), histotype (high-grade serous, low-grade serous, endometrioid, clear cell carcinoma, carcinosarcoma, mucinous, other/unknown), tumor stage (stage I–II, III, IV, unknown/missing), chemotherapy and surgery sequence (adjuvant, neoadjuvant, surgery without chemotherapy, chemotherapy without surgery, missing sequence, no surgery/chemotherapy), Charlson-Deyo Comorbidity Index (0, 1, ≥2), year of diagnosis (2012–2015, 2016–2019), insurance status (yes, no), treatment facility type (academic/research, non-academic), the distance of the facility from a patient’s residential address at diagnosis, location of facility (Northeast, South, Midwest, West), neighborhood education level, and median household income. Neighborhood education level was documented from the American Community Survey based on patient ZIP code, categorized into quartiles of adult ≥25 years who did not graduate from high school (≥17.6%, 10.9–17.5%, 6.3–10.8%, and <6.3%), which we combined into two groups at the median value (<10.9% as high education level and ≥10.9% as the low education level). Median household income of the ZIP code of residence was documented in the NCDB as estimated by the American Community Survey 2012–2016 and categorized into quartiles (<$40,227, $40,227–$50,353, $50,354–$63,332, and ≥$63,333), which we combined into two groups at the median value (≥$50,353 and <$50,353).

### Statistical analysis

Descriptive characteristics by receipt of targeted therapy were calculated as mean and standard deviation (SD) for continuous variables and frequency and percent for categorical variables. We explored the use of targeted therapy over time by the four racial and ethnic groups by computing the proportion of ovarian cancer patients receiving targeted therapy in each year of the study period. We used univariate and multivariable-adjusted logistic regression to compute the odds ratios (OR) and 95% confidence intervals (CI) associating the different factors of interest with receipt of targeted therapy. Multivariable models included all potential factors associated with receipt of targeted therapy.

Additionally, we explored the timing of targeted therapy in relation to receipt of surgery and considered receipt of targeted therapy as part of neoadjuvant therapy or adjuvant therapy. Neoadjuvant targeted therapy was defined as initiation of targeted therapy >21 days but ≤180 days before interval debulking surgery. Adjuvant targeted therapy was defined as the initiation of targeted therapy between 1–120 days after primary debulking surgery. Using this definition, we descriptively characterized the proportion of patients receiving neoadjuvant *vs*. adjuvant targeted therapy by race and ethnicity. All statistical analyses were performed with SAS version 9.4 (SAS Institute Carey, NC).

## Results

The total study population included 99,286 patients diagnosed with stage I–IV ovarian cancer between 2012 and 2019, of whom 4,029 (4.1%) received targeted therapy (**Table 1**). Ovarian cancer patients who received targeted therapy were less likely to be ≥80 years old compared with those who did not receive targeted therapy (5.3% *vs*. 9.1%). Patients who received targeted therapy were more likely to be diagnosed between 2016 and 2019 (78% *vs*. 48%), more likely to be diagnosed with stage III (47% *vs.* 36%) or stage IV disease (and 40% *vs*. 26%), receive neoadjuvant chemotherapy (33% *vs*. 14%), be diagnosed with other/unknown histology (65% *vs*. 8%), have unknown grade (72% *vs*. 50%), live in neighborhoods with a high education level (58% *vs*. 55%), and have health insurance (97% *vs*. 92%) compared with patients who did not receive targeted therapy.

**Table 1:** Baseline characteristics of the cohort by receipt of targeted therapy and odds ratios (ORs) and 95% confidence intervals (CIs) associating the different demographic and clinical characteristics with receipt of targeted therapy among ovarian cancer patients identified from the National Cancer Database 2012–2019 (N=99,286).

		Targeted therapy	No targeted therapy	Univariate analysis	Multivariable analysis	
		N=4,029 (4.1%)	N= 95,257 (95%)	OR (95% CI)	OR (95% CI)	
Age at diagnosis, years	<50	598 (15)	17,392 (21)	Ref	Ref	
	50–65	1,837 (46)	37,564 (42)	1.42 (1.30–1.56)	1.03 (0.91–1.17)	
	66–79	1,381 (34)	29,153 (28)	1.38 (1.25–1.52)	0.88 (0.77–1.00)	
	≥80	213 (5.3)	11,148 (9.1)	0.56 (0.48–0.65)	0.55 (0.45–0.67)	
	Continuous (mean and SD)	61.7 (12)	62.0 (15)	0.99 (0.99–1.00)	0.99 (0.98–0.99)	
Race and ethnicity	NHW	3,226 (80)	74,966 (79)	Ref	Ref	
	NHB	367 (9.1)	9,248 (9.7)	0.92 (0.83–1.03)	0.87 (0.76–1.00)	
	Asian	174 (4.3)	4,265 (4.5)	0.95 (0.81–1.11)	0.97 (0.80–1.16)	
	Hispanic	262 (6.5)	6,778 (7.1)	0.90 (0.79–1.02)	0.92 (0.79–1.08)	
Tumor Stage	I–II	261(6.5)	28,529 (30)	Ref	Ref	
	III	1898 (47)	34,042 (36)	6.09 (5.35–6.94)	4.64 (3.95–5.46)	
	IV	1615 (40)	24,412 (26)	7.23 (6.34-8.25)	6.05 (5.11–7.17)	
	Unknown	255 (6.3)	8,274 (8.7)	3.37 (2.83–4.01)	3.61 (2.94–4.43)	
Histology	High-grade serous	840 (21)	27,706 (29)	Ref	Ref	
	Low-grade serous	33 (0.8)	1,377 (1.5)	0.79 (0.56–1.12)	1.16 (0.74–1.82)	
	Endometrioid	69 (1.7)	6,177 (6.5)	0.37 (0.29–0.47)	1.00 (0.75–1.32)	
	Clear cell carcinoma	218 (5.4)	5,834 (6.1)	1.23 (1.06–1.43)	1.93 (1.61–2.31)	
	Carcinosarcoma	155 (3.9)	3,348 (3.5)	1.53 (1.28–1.82)	1.42 (1.16–1.74)	
	Mucinous	102 (2.5)	5,303 (5.6)	0.63 (0.52–0.78)	2.04 (1.57–2.64)	
	Other/unknown	2612 (65)	45,512 (48)	1.89 (1.75–2.05)	1.97 (1.79–2.17)	
Chemotherapy and surgery sequence	Adjuvant	1,751 (43)	40,563 (43)	Ref	Ref	
	Neoadjuvant	1340 (33)	13,755 (14)	2.26 (2.10–2.43)	1.26 (1.15–1.38)	
	Surgery without chemotherapy	23 (0.6)	16,398(17)	0.03 (0.02–0.05)	0.04 (0.02–0.07)	
	Chemotherapy without surgery	650 (16)	10,999 (12)	1.37 (1.25–1.50)	0.75 (0.67–0.85)	
	Surgery plus chemotherapy with missing sequence	17 (0.4)	9,696 (10)	1.50 (1.30–1.71)	1.24 (1.06–1.45)	
	No surgery or chemotherapy	248 (6.2)	3,846 (4.0)	0.04 (0.03–0.07)	0.02 (0.01–0.04)	
Charlson/Deyo comorbidity score	0	3,215 (80)	74,933 (79)	Ref	Ref	
	Charlson/Deyo comorbidity score	1	600 (15)	14,198 (15)	0.99 (0.90–1.08)	1.02 (0.92–1.13)
		≥2	214 (5.3)	6,126 (6.4)	0.81 (0.71–0.94)	0.78 (0.66–0.93)
Year of Diagnosis	2016–2019	3,160 (78)	45,761 (48)	Ref	Ref	
	2012–2015	869 (22)	49,496 (52)	0.25 (0.24–0.27)	0.29 (0.26–0.31)	
Insurance	Yes	3,867 (97)	90,225 (92)	Ref	Ref	
	No	112 (2.8)	3,573 (3.8)	0.73 (0.61–0.89)	0.84 (0.67–1.05)	
Hospital Type	Academic	1551 (40)	34,731 (39)	Ref	Ref	
	Community	2,298 (60)	53,828 (61)	0.96 (0.90–1.02)	1.01 (0.93–1.09)	
Distance (miles) to facility	<5.8	1,414 (35)	34,313 (36)	Ref	Ref	
	5.8–13.4	902 (22)	21,945 (23)	0.99 (0.92–1.09)	1.05 (0.95–1.17)	
	13.5–38.6	971 (24)	22,353 (24)	1.05 (0.97–1.15)	1.05 (0.94–1.16)	
	≥38.7	742 (18)	16,646 (18)	1.08 (0.99–1.18)	1.06 (0.94–1.19)	
Region	West	581 (15)	16,613 (19)	Ref	Ref	
	Northeast	786 (20)	18,473 (21)	1.22 (1.09–1.36)	1.18 (1.04–1.34)	
	Midwest	916 (24)	21,129 (24)	1.24 (1.12–1.38)	1.24 (1.10–1.41)	
	South	1,567 (41)	32,344 (37)	1.39 (1.26–1.53)	1.47 (1.32–1.65)	
Neighborhood education level	<10.9% NHD	1,978 (58)	44,942 (55)	Ref	Ref	
	≥10.9% NHD	1,424 (42)	35,573 (46)	0.86 (0.80–0.92)	0.86 (0.79–0.95)	
Household Income	≥$50,353	2,169 (64)	50,723 (62)	Ref	Ref	
	<$50,353	1,222 (36)	31,669 (38)	0.90 (0.84–0.97)	0.95 (0.87–1.05)	

Count and proportions are reported for categorical variables and mean, and standard deviation is reported for the continuous variable of age at diagnosis. NHD, No high school degree; OR, Odds Ratio; CI, Confidence Interval.

**Figure 1:**
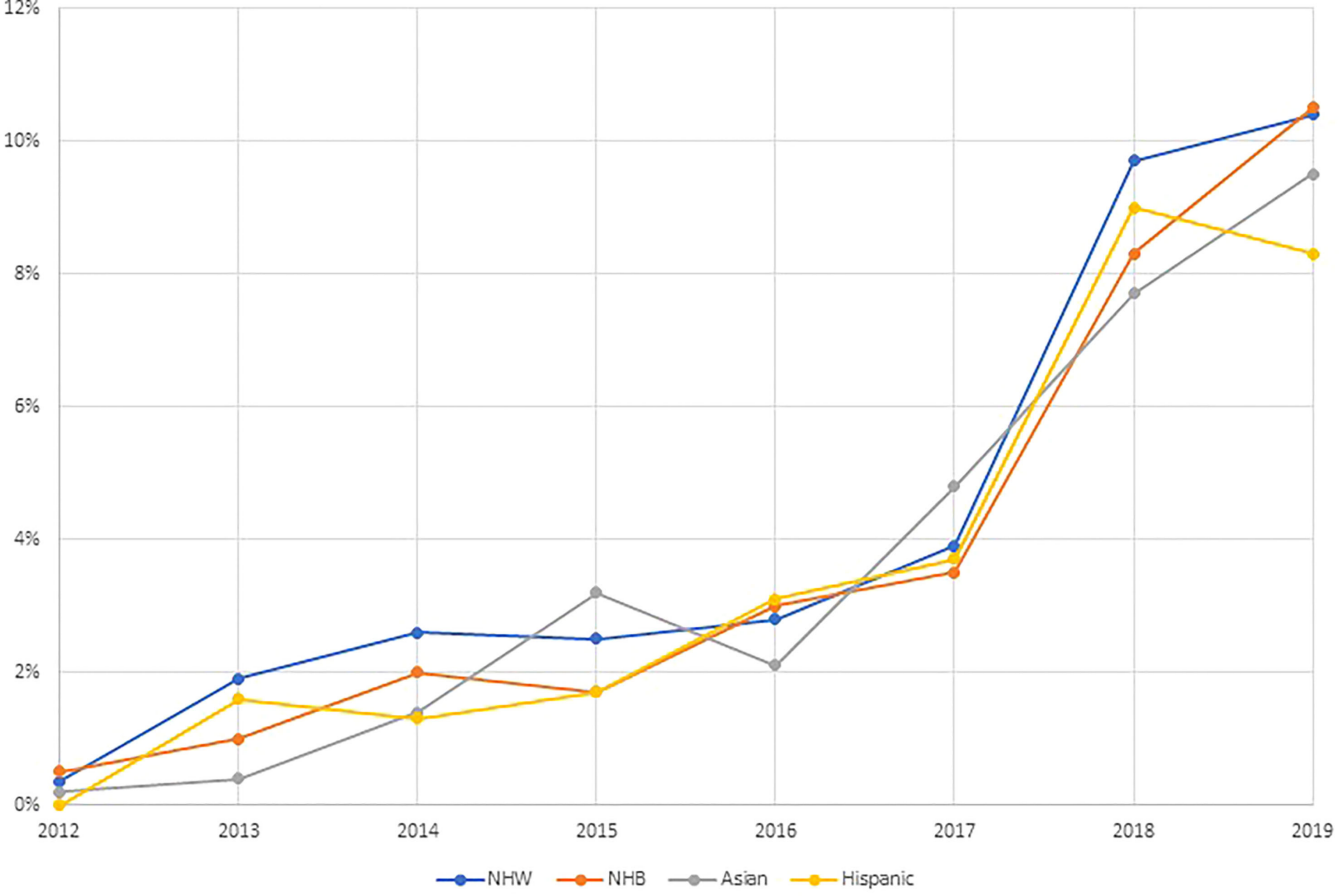
Trends in the receipt of targeted therapies by race and ethnicity among ovarian cancer patients identified from the National Cancer Database 2012–2019 (N=99,286).

In **Figure 1**, we illustrate the proportion of ovarian cancer patients receiving targeted therapy each year of the study period by race and ethnicity. In 2012, less than 1% of all patients received targeted therapies. The proportion of patients receiving targeted therapy rose to approximately 3% by 2017, with NHW patients slightly more likely to receive targeted therapy in this time period. In 2018, there was a sharp increase in the proportion receiving targeted therapy, with approximately 10% of all ovarian cancer patients receiving targeted therapy. In general, the proportion receiving targeted therapy over time did not vary meaningfully across racial and ethnic groups.

In **Table 1** we also present the univariate and multivariable-adjusted models associating the factors of interest with receipt of targeted therapy. In the multivariable-adjusted logistic regression analysis, we observed that patients diagnosed at 66–79 years of age or ≥80 years of age were less likely to receive targeted therapy than those diagnosed <50 years (OR=0.88, 95% CI: 0.77–1.00 and OR=0.55, 95% CI: 0.45–0.67, respectively). NHB ovarian cancer patients were less likely to receive targeted therapy than NHW women (OR=0.87, 95% CI: 0.76–1.00). As expected, receipt of targeted therapy increased over time and ovarian cancer patients diagnosed 2012–2015 were less likely to receive targeted therapy compared with those diagnosed 2016–2019 (OR=0.29, 95% CI: 0.26–0.31). Also as expected, patients diagnosed with stage III or IV disease were more likely to receive targeted therapy compared with those who were diagnosed with stage I/II disease (OR=4.64, 95% CI: 3.95–5.56 and OR=6.05, 95% CI: 5.11–7.17, respectively). Interestingly, those treated with neoadjuvant chemotherapy were more likely to receive targeted therapy anytime in their treatment course than those who received adjuvant therapy (OR=1.26, 95% CI: 1.15–1.38). Receipt of targeted therapy was more common among women diagnosed with clear cell carcinoma (OR=1.93, 95% CI: 1.61–2.31), carcinosarcoma (OR=1.42, 95% CI: 1.16–1.74), and mucinous (OR=2.04, 95% CI: 1.57–2.64) histologies compared with women diagnosed with high-grade serous ovarian cancer. Ovarian cancer patients presenting with a comorbidity score ≥2 at the time of diagnosis were less likely to receive targeted therapy (OR=0.78, 95% CI: 0.66–0.93) compared with those without any underlying comorbidities. Residing in a neighborhood with a higher proportion of residents with less than high school education was associated with lower odds of receiving targeted therapy (OR = 0.86, 95% CI: 0.79–0.95) compared with those who resided in neighborhoods with higher educational attainment. Similarly, lack of health insurance was associated with lower odds of receiving targeted therapy (OR = 0.84, 95% CI: 0.67–1.05) compared with patients who had health insurance. We also observed variation in the use of targeted therapy by region of diagnosis, ovarian cancer patients diagnosed in the Northeast, Midwest, and South were more likely to receive targeted therapy than those diagnosed in the West (OR=1.18, 95%CI: 1.04–1.34, OR=1.24, 95% CI: 1.10–1.41, and OR=1.47, 95% CI: 1.32–1.65, respectively).

**Figure 2:**
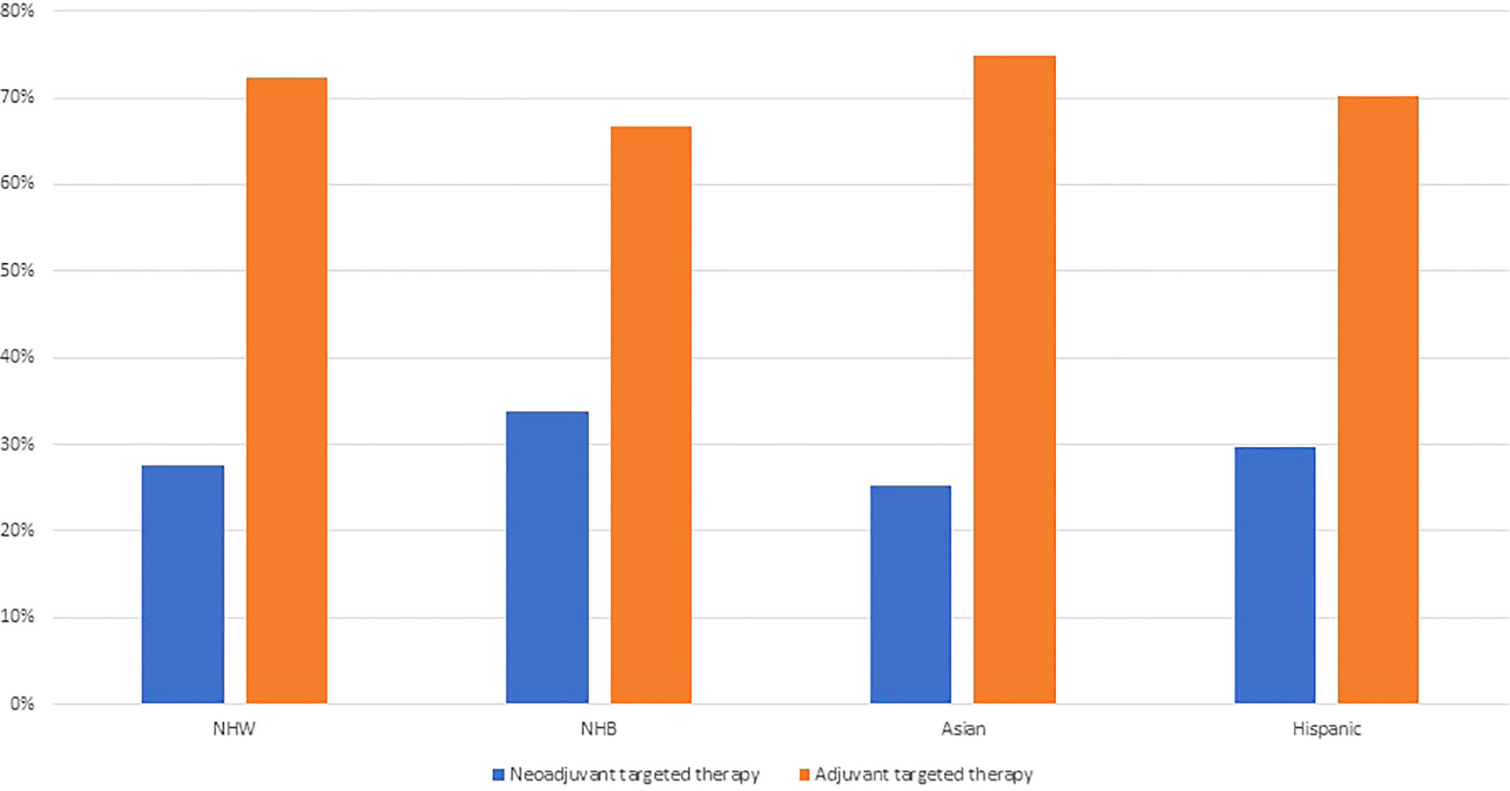
Receipt of targeted therapy by treatment sequence and racial and ethnic groups among ovarian cancer patients identified from the National Cancer Database 2012–2019 (N=99,286).

Based on our results demonstrating that patients who received targeted therapy were more likely to receive neoadjuvant chemotherapy, we explored treatment sequence in relation to the timing of receipt of targeted therapy by race and ethnicity. Interestingly, we report that among patients who received targeted therapy, 28% received neoadjuvant targeted therapy. Moreover, NHB ovarian cancer patients were more likely to receive neoadjuvant targeted therapy (34%), than NHW patients (28%), Asian patients (25%), or Hispanic patients (30%) (**Figure 2**).

## Discussion

In this study, we investigated factors associated with receipt of targeted therapy among ovarian cancer patients. We identified notable differences in receipt of targeted therapy by factors relating to indications for targeted therapy—including age at diagnosis, stage, and comorbidities present at diagnosis—as well as factors related to access to quality cancer care—including neighborhood education level and health insurance status. We also reported that although there we did not observe racial and ethnic disparities in receipt of targeted therapy over time, we did observe that NHB women were less likely to receive targeted therapy than NHW women, but were more likely to receive targeted therapy as part of neoadjuvant treatment, which is associated with an increased risk of complications that can delay or prevent surgery.

Although targeted therapies have been incorporated into clinical guidelines to treat ovarian cancer for approximately ten years, there have been few studies that have examined patient demographic and clinical characteristics associated with receipt of targeted therapy. In our study, we observed that only 4.1% received targeted therapy. This relatively low proportion of patients receiving targeted therapy may be explained by the limitations of use of some targeted therapies, especially bevacizumab, because they can worsen high blood pressure, are associated with an increased risk of bowel perforation, can worsen or cause bleeding, and may be cost prohibitive for some patients (2, 20). Several studies have reported that marginalized populations and individuals from lower socioeconomic status neighborhoods are less likely to receive ovarian cancer treatment in accordance with the NCCN clinical guidelines (21–25). In the current study, we observed that patients without health insurance and those that lived in neighborhoods with a high proportion with less than high school education were less likely to receive targeted therapy. These results are consistent with previous studies examining receipt of guideline care among ovarian cancer patients (22, 23). It has been previously reported that health care disparities tend to emerge or increase with the advent of new therapeutic approaches to cancer due to barriers in access and affordability of these new treatments (26). Therefore, additional studies examining receipt of targeted therapies among ovarian cancer patients are necessary to understand barriers in access to care and to achieve health equity.

In this study, we observed that a high proportion of ovarian cancer patients who received targeted therapy, received it prior to surgery. Current NCCN guidelines caution the use of targeted therapies, namely bevacizumab, in the neoadjuvant setting due to increased risk of complications that could delay or prevent surgery (2, 10, 14). Importantly, in the current study we observed that nearly 30% of patients who received targeted therapy, received targeted therapy in the neoadjuvant setting, and this was most common among NHB women. The high proportion of patients, especially NHB ovarian cancer patients, who received targeted therapy in the neoadjuvant setting may lead to increase in treatment-related complications, lower optimal debulking status, and worse survival. Bevacizumab may be administered in the neoadjuvant setting to treat ascites or pleural effusions, with the goal of improving the patient’s surgical candidacy (27, 28). Our results may suggest that NHB women are more likely to present with ascites or pleural effusions at the time of their cancer diagnosis, which may be worthwhile to explore in future studies. When we examined the proportion of patients receiving targeted therapy by race and ethnicity over the study time, we did not observe meaningful differences in receipt of targeted therapy by race and ethnicity over time. However, in our multivariable-adjusted models, NHB patients were less likely to receive targeted therapy than NHW patients. These potential racial and ethnic disparities in the use of targeted therapies, could lead to less favorable treatment outcomes and survival, which warrant further investigation in studies with detailed information regarding ovarian cancer treatment and outcomes.

This study has some important limitations. First, although our study population included all ovarian cancer patients diagnosed and reported to the NCDB, which covers approximately 70% of the population of the U.S., the results may not be generalizable to the total population of ovarian cancer patients in the U.S. as those treated at Commission on Cancer accredited facilities may differ from those treated at non-Commission on Cancer accredited facilities (29). Second, although we had information on receipt of targeted therapy, we did not have information on the type of targeted therapy. Bevacizumab was introduced into clinical guidelines as a potential agent to be included in chemotherapy regimens for ovarian cancer in 2012; however, other targeted therapies have also been approved as maintenance therapies. These include PARP inhibitors, which may be captured in the current ‘targeted therapy’ variable. Still, we expect that this would be a small proportion of therapies captured by this variable as PARP inhibitors were not approved for use until 2018 and are currently approved for use as a first-line maintenance therapy (30). Third, we were also limited in the ability to look at other racial and ethnic groups, or disaggregate the racial and ethnic groupings, due to the smaller sample sizes. Regardless, this study is the most extensive study investigating factors associated with receipt of targeted therapy among ovarian cancer patients.

In conclusion, our study results showed that there has been an overall increase in the use of targeted therapies in first line ovarian cancer treatment, with variation in use by important sociodemographic factors. We observed that NHB women may be less likely to receive targeted therapy than NHW women, and more likely to receive targeted therapy as part of neoadjuvant treatment. Our results warrant further evaluation of factors related to use and timing of targeted therapies in ovarian cancer treatment, especially in the context of cancer health disparities, and the possible impact on treatment effectiveness and survival in a cohort of patients with more detailed treatment information.

## Data availability statement

Publicly available datasets were analyzed in this study. This data can be found here: Data come from the publicly available National Cancer Database.

## Ethics statement

Current study was exempt from Institutional Review Board (IRB) review as the NCDB data is a de-identified data source. The ethics committee waived the requirement of written informed consent for participation.

## Author contributions

SA had full access to all the data in the study and take responsibility for the integrity of the data and the accuracy of the data analysis. Conception and design: SA, LC, EB, SS, and JS; financial support: SA, LC, and EB; administrative support: SA; provision of study materials or patients: SA; collection and assembly of data: SA, LC, EB, SS, and JS; data analysis and interpretation: SA, LC, EB, SS, JS, and JK; initial manuscript draft: LC, SA, and EB; manuscript editing: all authors; final approval of manuscript: all authors; accountable for all aspects of the work: all authors
